# Tribological Performance of Environmentally Friendly Bio-Degradable Lubricants Based on a Combination of Boric Acid and Bio-Based Oils

**DOI:** 10.3390/ma13173892

**Published:** 2020-09-03

**Authors:** Tomasz Trzepieciński

**Affiliations:** Department of Materials Forming and Processing, Rzeszow University of Technology, al. Powst. Warszawy 8, 35-959 Rzeszów, Poland; tomtrz@prz.edu.pl; Tel.: +48-17-743-2527

**Keywords:** coefficient of friction, deep drawing, mechanical engineering, sheet metal forming, steel sheet

## Abstract

Finding effective and environmentally friendly lubrication to use in sheet metal forming operations presents a substantial environmental and economic challenge to the automotive industry. This paper examines the effectiveness of different lubricants in the reduction of the coefficient of friction (COF) in the process of sheet metal forming of the low carbon steel sheets. These lubricants are based on a combination of boric acid (H_3_BO_3_) and edible vegetable oils, both of which are natural and environmentally friendly. To evaluate the friction characteristics of the lubricants in a forming operation, a strip drawing friction test is used. This test consisted in drawing a specimen in the form of a sheet metal strip between two non-rotating counter-samples with radii of 200 and 10 mm. The effectiveness of environmentally friendly lubricants in reducing the COF was compared to the traditional petroleum-based lubricants which are used in sheet metal-forming operations. The effect of lubricant conditions and tool surface roughness on the value of COFs is studied. It was found that palm oil in both configurations of countersample radius, both as pure oil and with the addition of 5 wt.% of H_3_BO_3_, was the most effective in lowering the coefficient of friction. In most of the conditions analysed, the addition of boric acid into vegetable oils leads to an increase in the lubrication efficiency by up to 15% compared to pure oils. The effectiveness of lubrication by olive and rapeseed oils in decreasing the frictional resistances clearly depends on the nominal pressure applied.

## 1. Introduction

The friction occurring between a deformed sheet and the tool surface has a significant impact on the course of plastic deformation, tool life and product performance. In sheet metal forming (SMF), friction forces also decide on both the value and state of stress in the surface layer of the material being deformed. Generally, it can be stated that the effects of interaction of friction forces in the sheet metal forming processes are almost always unfavourable. Therefore, the basic issue in the SMF is to reduce frictional resistance through the use of carefully selected lubricants and lubrication methods [1,2,3].

The friction phenomenon occurring in SMF significantly differs from the friction occurring in the kinematic joints of machines due to such factors as: (i) high unit pressure, (ii) low relative speeds of displacement of the surfaces in contact, (iii) continuous change of the friction surfaces, (iv) the different roles and functions of the lubricants. The unit pressures occurring on the contact surface are very large and can significantly exceed the value of yield stress of one workpiece material [4,5]. As a result of high pressures, the friction conditions change significantly because of decreases in lubrication efficiency, causing a break in the lubricating film and leading to direct contact of the asperities of rough surfaces. External friction or, more precisely, a set of tribological processes occurring in the contact zone of the deformed metal-tool determines both the state of the surface layer of the product and changes in the state of the surface layer of the tool [6,7,8].

Lubrication depends on the introduction of a third body (lubricant) between the surfaces in contact. Lubricants have the form of fluid, solid and aerosol substances which, introduced into the friction interface, reduce frictional resistance and allow the change of friction mechanism from dry friction to a mixed lubrication regime [9]. The purpose of using lubricants is to reduce the energy losses necessary to overcome friction and reduce the intensity of tool wear [10,11]. During the deformation process, the lubricant influences the change of surface topography of the workpiece, the character of flow of the deformed metal, decreases unit pressure and the coefficient of friction (COF), and also improves the quality of the product surface [12,13,14]. Increasing the efficiency of lubrication in the SMF can be obtained by texturing the tool surface, thus creating so-called ‘oil pockets’, which are a reservoir for the lubricant. Lubricants used in plastic working must fulfil the following requirements: good lubricating properties, stability of properties, good wettability and adhesion to surfaces, corrosion prevention and effective removal of the heat emitted as a result of friction.

An increase in the cost of petroleum-based lubricants and environmental law regulations has caused vegetable oils to become increasingly attractive in a wide range of metal-forming applications [15,16,17]. Sunflower, coconut, soya bean, peanut, linseed, and rapeseed oils are considered as potential bio lubricants [18]. Lubricating oil additives can be classified as dispersants, antioxidants, and anti-wear [19]. One of the approaches to reducing friction in a boundary lubrication regime is the use of a dilute solution of saturated fatty acids (palmitic and stearic acid) in vegetable oils [20,21,22]. Cortes et al. [23] evaluated the tribological characteristics of sunflower oil modified with titanium dioxide and silicon dioxide nanoparticles as lubricant additives. It was found that the rheological behavior of the sunflower-based lubricant is dependent on the type and concentration of nanoparticles. Bhaumil et al. [24] studied the tribological performance of cashew nut shell oil with the use a graphene oxide additive. The friction modifiers from the cashew nut shells were tested on a four-ball tribometer and the results show that they act as effective extreme-pressure additives at higher loads.

The correlations between the lubrication performance, physicochemical properties and molecular structure of bio-based lubricants were reviewed by Syahir et al. [25]. It was concluded that natural oils are a promising substitute for various applications. State-of-the-art, eco-friendly nanoparticles for improving the tribological properties in conventional lubricants have been presented by Peña-Parás et al. [26].

When compared to mineral and synthetic oils, vegetable oils have a number of advantages including lower volatility, higher shear stability, higher lubricity and a substantially higher viscosity index [18]. Moreover, natural oils have a lower tendency to react with most of the metals susceptible to oxidation and corrosion. A general comparison of the properties of mineral and vegetable oils is listed in Table 1.

The advantages and disadvantages of bio-lubricants and the potential of vegetable oil-based lubricants for automotive applications were discussed by Mobarak et al. [27]. A wide range of state-of-the-art reviews have shown that environmentally friendly lubricants are potential alternative lubricants because of their high load-carrying abilities, low COF values, high ignition temperature, low evaporation rates, excellent anti-wear characteristics, high viscosity index, low emissions into the atmosphere, and non-toxicity for humans [27].

In recent decades, a great emphasis has been placed on ecological aspects in industrial practice. Therefore, the environmental sustainability of SMF is nowadays considered urgent. The development of environmentally friendly lubricants has become one of the main issues for technologists in the design of SMF processes. Reviewing the literature, there have been several studies related to the development of eco-friendly lubricants in the past two decades. Mohammed [28] explored the lubricating properties of date palm fruit syrup by using a ball-on-disc tribological test. The palm fruit syrup was found to be very effective as an additive to deionised water or as 100% syrup in reducing the friction when compared to dry sliding. Good tribological properties can be attributed to the presence of fatty acids such as palmitic acid, oleic acid and linoleic acid. Tahir et al. [29] also tested the tribological properties of date palm fruit syrup in a pin-on-disc test. Abdulqadir et al. [30] tested the possibility of applying various vegetable oils such as black soap, ground nut oil, sheabutter oil and red palm oil for SMF processes. It was found that the most effective in reducing the COF was red palm oil. Bahari et al. [31] found that addition of zinc dialkyl dithiophosphate to soybean oil reduced wear by 57% compared to its state as pure oil. Kapok oil is found to be very effective in reducing friction in the pin-on-disc test as compared to mineral oil SAE 20W40 and palm oil [32]. Ming et at. [33], Sridhara and Satapathy [34], Cornelio et al. [35] and Zhang et al. [36] have explored different strategies, such as adding environmentally friendly nano-fillers as additives to the base oil to reduce the frictional resistance. On the other hand, Chauhan and Chhibber [37] and Ruggiero et al. [38,39] analysed the tribological performance of water-based lubricants with an extract from vegetable oils. The lubrication ability of rapeseed oils containing various concentrations of nanoparticle additives (copper oxide and silica) was investigated by Zareh-Desari and Davoodi [40] in a ring compression test. The authors concluded that compared to soybean oil, rapeseed oil has a superior lubricating ability which could be a contribution of its lower degree of unsaturation and the longer length of its hydrocarbon chains. Carcel et al. [41] investigated the frictional properties of pure zinc or galv-annealed coated steel sheets lubricated using vegetable oils that simulate similar tribological conditions to those which reign in the stamping process. Ameen et al. [42] studied the tribological properties of AlSn20Cu alloy in pin-on-disc apparatus with the use of a mixture of soybean oil and used frying oil (methyl esters of fatty acids). The boundary and/or mixed lubrication performance of the mixture used gives very promising results regarding the possibility of decreasing the wear and frictional resistances. Moreover, with the addition of additives, it is possible to increase the performance characteristics of the lubricant tested. Taha-Tijerina et al. [43] evaluated the tribological performance of corn, sunflower, soybean and paraffinic oils reinforced with silica nanoparticles. The experimental tests using a four-ball universal tribotester showed that the addition of SiO_2_, even in small concentrations, resulted in a significant improvement in the load-carrying capacity. Investigations on the effect of Al_2_O_3_ nanoparticle additives on the lubrication performance in the deep drawing process were conducted by Zareh-Desan [44]. The results showed that the capacity for reducing the friction of the base oil was enhanced by adding the nanoparticles, which improves the surface quality of the products and reduces the required forming load.

A review conducted by Uflyand et al. [45] was focused on the effect of metal-containing nanomaterials (metal sulphides, metal oxides, rare-earth compounds and nanocomposites) on tribological performance in oil lubrication. The authors concluded that the tribological mechanisms of nanolubricants should be studied and examined in more detail. Karmakar et al. [46] studied the use of chemically modified natural oils and additives to prepare environmentally friendly components of lubricants. It has been concluded that environmentally friendly lubricants are emerging as future green products because these bio-degradable lubricants have a better wear performance and higher viscosity index than other lubricants. Ingarao et al. [47] gives an overview of the main topics concerning SMF problems related to resource efficiency including tribological aspects of the use of environmentally friendly lubricants.

Most tribological tests used to characterise the performance of ecolubricants are carried out using a pin-on-disc tribometer. The infinite friction path and continuous repeatability of contact of the pin and the specimen in this test does not reflect the conditions prevailing in the sheet metal forming process. The infinite friction track in the pin-on-disc tribometer causes a dramatic intensification of the wear which is very limited during the SMF of drawpieces. Experimental investigations of the ecolubricants under conditions corresponding to SMF have not received great attention in the literature. To address these challenges, the present study focuses on the experimental testing of ecolubricants based on a combination of boric acid and different vegetable oils. As a tribological test, the strip drawing test has been selected to represent the contact conditions in SMF. The aim of friction testing was to show the effect of lubricant conditions, tool surface roughness and strip sheet orientation on the value of COFs when testing the carbon steel sheets. The effectiveness of the reduction of the COF by environmentally friendly lubricants was compared to traditional mineral lubricants.

## 2. Materials and Methods

### 2.1. Material

As a test material, a 0.8-mm-thick low-carbon steel sheet (DC04 according to the EN 10130: 2009 standard) manufactured in a cold rolling process was used. The required chemical composition of uncoated DC04 steel sheet consists of EN10130:2009 [48] standard as ≤0.08% C, ≤0.4% Mn, ≤0.03% P, ≤0.03% S, with the remainder as Fe. DC04 material is a deep-drawing grade of steel and is characterised by very good formability. Due to their mechanical properties, especially a high susceptibility to the work hardening phenomenon, DC04 grade is suitable for automobile bodies, floor panels, side panels, drums, and the products of the domestic appliance sector where high ductility and strength are required. Uncoated cold rolled steels for cold forming are suitable for contact with food under certain conditions laid down in Regulation (EC) 1935/2004 [49,50].

The mechanical properties of sheet metals (Table 2) were determined in a uniaxial tensile test in a Z100 machine (Zwick/Roell, Ulm, Germany) carried out at room temperature according to EN ISO 6892-1 [51]. The value of work hardening exponent of a material is a measure of how quickly the material gains strength when it is being deformed. The work hardening exponent can be simply obtained from the slope of the true stress vs. true strain curve in a uniaxial tensile test plotted on a logarithmic scale. The simplest model for work hardening is known as the Ludwik/Holloman equation:

(1) $$\sigma_{p}=K\times \varepsilon^{n}$$

where *σ_p_* is stress, *K* is strength coefficient and *ε* is plastic strain.

Strip specimens for tensile tests were cut from the sheet metal in three directions: at an angle of 0°, 45° and 90° with respect to the rolling direction of the sheet metal. Three specimens were cut for each direction and average values of mechanical parameters were determined.

The basic surface roughness parameters of the steel sheet tested have been determined using a Talysurf CCI Lite 3D optical profiler (Taylor Hobson, Leicester, Great Britain). Table 3 lists the selected surface roughness parameters determined: average roughness *Sa,* skewness *Ssk*, kurtosis *Sku*, root mean square roughness *Sq,* maximum pit depth *Sv*, 10-point peak-valley surface roughness *Sz*, root mean square gradient *Sdq*, total height *St*, highest peak of the surface *Sp*, and developed interfacial area ratio *Sdr*.

**Figure 1:**
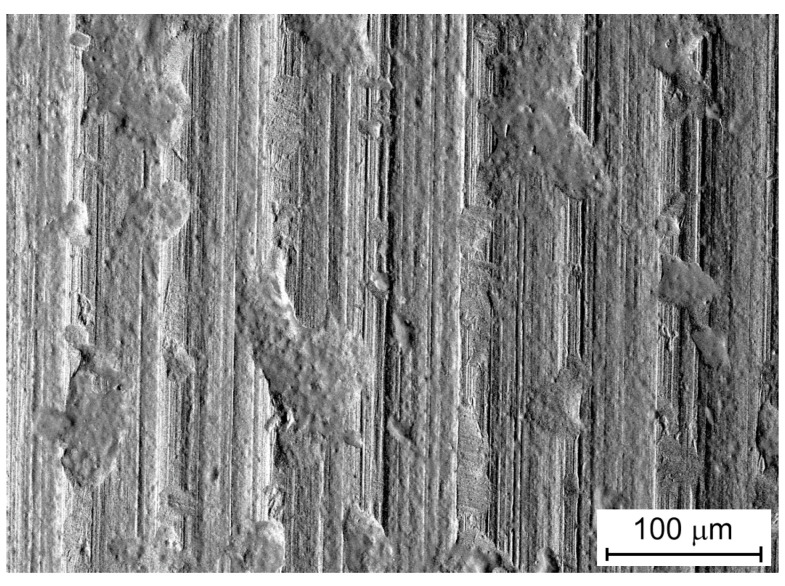
The SEM micrograph of original surface, magnification ×530.

The SEM micrograph of the original surface of the DC04 sheet metal reveals the existence of iron oxides (Figure 1) formed in the rolling process. The surface exhibit a directional topography (Figure 1) resulted from the rolling process.

### 2.2. Strip Drawing Test

**Figure 2:**
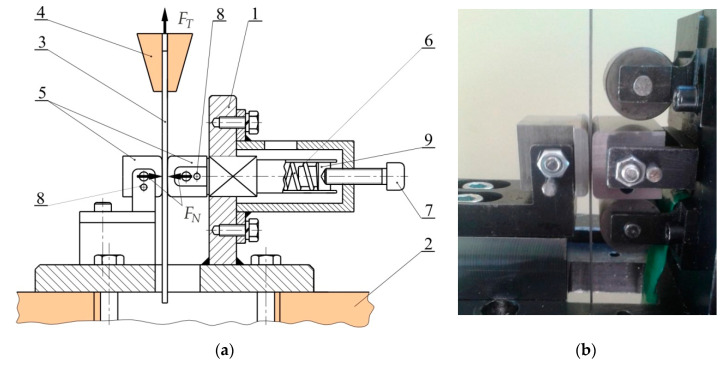
(**a**) layout and view of the device and (**b**) view of the working zone: 1—frame, 2—bottom grip of testing machine, 3—specimen, 4—upper grip of testing machine, 5—countersamples, 6—spring, 7—bolt; 8—fixing pins, 9—teflon washer.

Assessment of the tribological performance of the steel sheets under different friction conditions has been carried out using the strip drawing test which is commonly used to represent contact conditions in SMF. The test consists in drawing a specimen in the form of a sheet metal strip between two non-rotating counter-samples with a cylindrical surface. The strips for the friction test of approximately *l* = 400 mm long and *b* =18 mm wide, were cut from the sheet metal along its rolling direction. A schematic diagram of the specially designed friction simulator is shown in Figure 2.

Frame 1 of the device was mounted on the bottom grip 2 of the universal tensile testing machine. Tests were carried out with different countersample 5 radii to ensure a wide range of normal pressures: countersamples with a radius of *R* = 200 mm (Figure 2), cylindrical countersamples with a radius of *R* = 10 mm. After placing the specimen between the counter-samples and clamping it in the upper grip of the testing machine, the minimum required value of the normal force was applied. The value of the normal force was increased gradually during the displacement of the specimen in the device. Computer equipment was used to record the changes in the values of both the normal force and the pulling force during this process.

The countersamples were made of cold-work tool steel. The assumption adopted during the planning of investigations was to carry them out using countersamples with different roughnesses. The tests at small nominal pressures were carried out using countersamples with a radius *R* = 200 mm whose roughness parameters are shown in Table 4. The tests at higher pressures were carried out using rollers with a radius of *R* = 10 mm. These rollers were manufactured by the turning and grinding processes, so it was convenient to determine the performance of rollers with a surface roughness measured along the generating line of the rolls. In a later part of the article, the rolls will be identified by the arithmetic mean values which were 0.32, 0.63 and 1.25 μm. Moreover, the 3D surface roughness parameters (Table 5) were measured using an InfiniteFocus (Alicona Imaging GmbH, Raaba, Austria) measuring device.

The value of the friction coefficient μ in SDT is determined based on the relationship:

(2) $$\mu =\frac{F_{T}}{2F_{N}}$$

where: *F_T_* is the friction force and *F_N_*–normal (pressure) force.

The advantage of the device is the uncomplicated measurement of force parameters. Friction force *F_T_* is measured through the load cell of the Z100 testing machine (Zwick/Roell, Ulm, Germany) and is registered by the testing software testXpert® (Zwick/Roell, Ulm, Germany). This software is an integral part of the Z100 testing machine. The value of the normal force *F_N_* was constantly recorded using the electric resistance strain gauge technique, 4-Channel C-series strain/bridge input module NI 9237 (National Instruments, Austin, TX, USA) and Lab View DAQ software (National Instruments, Austin, TX, USA). Both force values were registered with a frequency of 50 Hz.

The pressure on the countersamples was applied by the spring *6* and screw *7*. The relationship between the force and the displacement of the springback in the range of spring deflection between 1 and 12 mm has been determined using a MultiTest 10-i testing machine (Mecmesin Ltd., Slinfold, Great Britain) and it was almost linear (R^2^ = 0.989). The normal force exerted on the specimen has been obtained by suitable deflection of the springback. The deflection of the springback has been realised by rotation of the bolt head knowing that the lead of the metric thread is equal to *P* = 1.25 mm. The normal force has been converted in nominal pressure *p_H_* using the relationship:

(3) $$p_{H}=\sqrt{\frac{0,418^{2}\;F_{N}\cdot E}{b\cdot R}}$$

where *F_N_* is the clamping force of the countersamples, *E* (205 GPa) is the Young’s modulus of the sheet material, *b* is the width of the specimen.

The range of pressures analysed is between 1 and 60 MPa to reproduce the evolutions of pressure encountered under the blankholder in SMF [52]. Due to the limitations of the friction simulator in reproducing such a wide range of pressures, two sets of countersamples were used with a radius of *R* = 200 mm and *R* = 10 mm for the range of pressures between 1 and 10 MPa and between 10 and 60 MPa, respectively.

Prior to the friction evaluates all specimens were degreased using acetone. The friction tests were conducted for two friction conditions: dry and lubricated. All experiments were carried out at a room temperature of 25 °C and constant speed of 10 mm·s^−1^ [12].

### 2.3. Lubricants

To compare the tribological performance of environmentally friendly lubricants, two main groups of lubricants were used. The first group consisted of the oils commonly used in SMF for different duty conditions.

- Machine oil (MO) LAN-46 (Orlen Oil, Kraków, Poland);
- Deep-drawing oil (DDO) L (Orlen Oil, Kraków, Poland);
- Heavy-Draw oil (HDO) 1150 (Lamson Oil, Rockford, IL, USA).

The properties of oils provided by the manufacturer are listed in Table 6.

The second group of lubricant consisted of edible vegetable oils (Table 7) with and without additives. All the vegetable oils used consist different composition of the following acids: palmitic acid (C16:0, 4–41%), stearic acid (C18:0, 1–6%), palmitoleic acid (C16:1, 0.1–1.8%), oleic acid (C18:1, 19–71%), linoleic acid (C18:2, 8–68%) and α-linoleic acid (C18:3, 0.2–56%). Oleic acid and linoleic acid predominate in the triglycerides of the vegetable oils used, such as sunflower, linseed, palm, and soybean [46].

The use of boric acid (BA) H_3_BO_3,_ purchased from Warchem (Marki, Poland), was investigated as a possible additive. The basic physical properties of BA are as follows: molecular mass-61.83 g·mol^−1^, density-1.44 g·cm^−3^, boiling point –30 °C and melting point –160 °C. The additive was dissolved in vegetable oils at a concentration wt. 5% [15,53]. The agitating process takes around half an hour to ensure that the particles suspended in the oils evenly. Prior to testing, both sides of the specimen were oiled by a roller system that permits one to obtain a uniform oil coating between 1.5 and 2 g·m^−2^, which is comparable with the conditions of the stamping process [41].

## 3. Results and Discussion

### 3.1. Effect of Nominal Pressure

**Figure 3:**
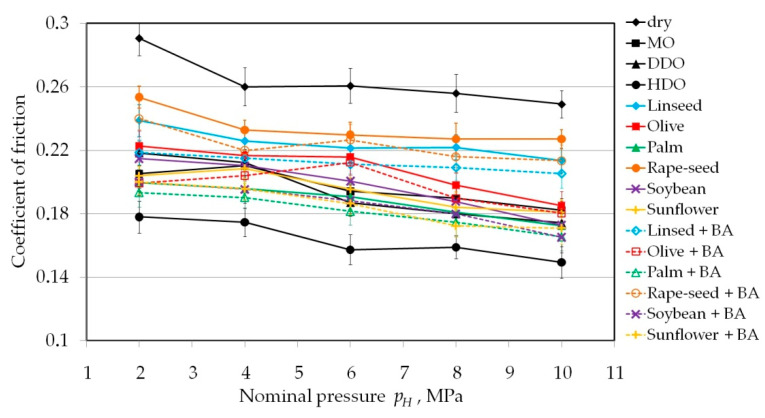
The effect of nominal pressure in the range between 2 and 10 MPa on the value of COF.

Figure 3 shows the change in the value of the COF depending on the value of the nominal pressure for rollers with a radius *R* = 200 mm. During the friction test, the value of the friction force was continuously recorded in such a way that for each pressure level a data set containing about 500 numerical values of the friction force was obtained. This permitted the determination of the average value of the COF for each specific nominal pressure (Figure 3). There is a clear tendency for the COF value to decrease with increasing pressure. This reduction is within the standard deviation and, although it is not very statistically significant, it has already been observed by Trzepiecinski and Fejkiel [12] during the strip drawing test with cylindrical counter-samples. The non-linear relationship between friction force and clamping (normal) force means that the COF is not constant beyond a certain load. It is also coincides with the results of Kirkhorn et al. [54]. Among deep-drawing quality oils, HDQ oil showed the greatest ability to reduce frictional resistance.

The vegetable oil that provided the greatest reduction in the COF is palm oil in configurations both as pure oil and with the inclusion of BA particles. Of all the vegetable oils used, palm oil contains the most compounds of palmitic acid (C16:0) and oleic acid (C18:1). Both linseed and rapeseed oils are characterised by the lowest ability to reduce frictional resistance.

**Figure 4:**
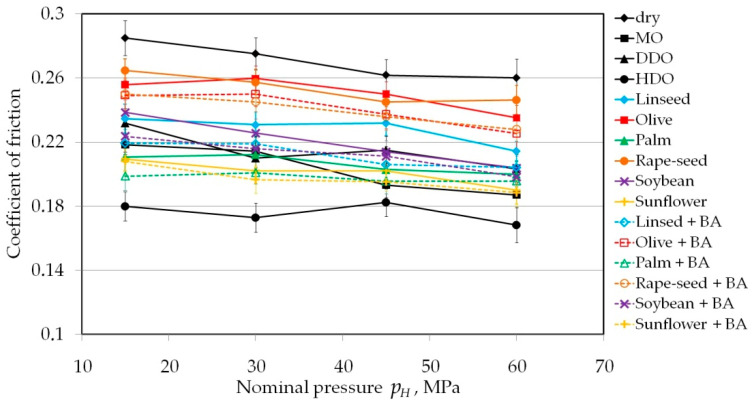
The effect of nominal pressure in the range between 15 and 60 MPa on the value of COF; countersample with roughness value of *Ra* = 0.32 mm.

Friction tests carried out with cylindrical countersamples with a radius of *R* = 10 mm showed a similar trend in the decrease of COF with pressure (Figure 4). HDO oil provided the best lubricating properties. In addition, the tendency for the COF to change value of during lubrication was the most stable with this oil over a wide range of values of applied pressure. Olive and rapeseed oils are the most effective vegetable lubricants. MO and DDO mineral lubricants qualitatively reduced friction to the same level as palm and sunflower oils. The above conclusions can be referred to tests conducted with rolls with roughness values of *Ra* = 0.63 and 1.25 μm. An accurate qualitative comparison of the friction test results will be presented in the following subsections.

**Figure 5:**
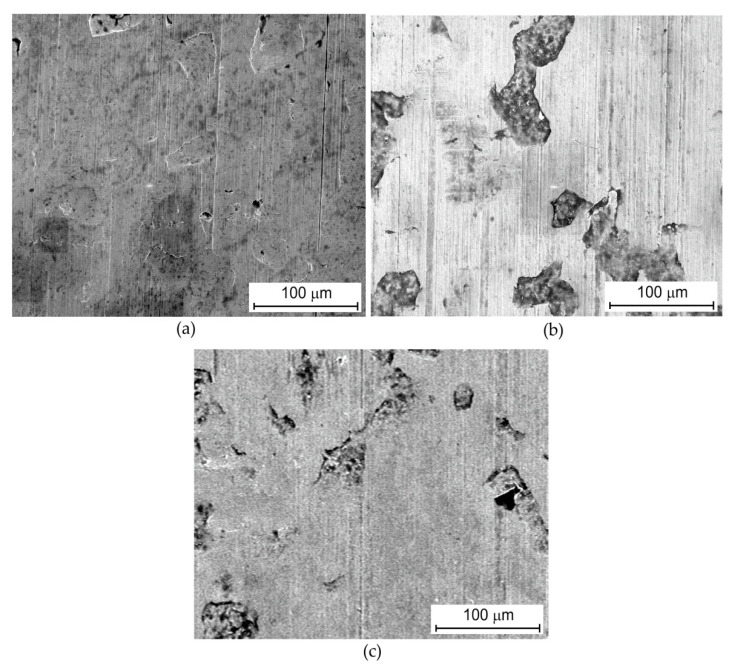
SEM micrographs of the sheet surface after friction tests carried out under following conditions: (**a**) dry friction, Ra = 0.32 μm, nominal pressure *p_H_* = 30 MPa (magnification ×800); (**b**) olive, *Ra* = 0.32 μm, *p_H_* = 30 MPa (magnification ×800) and (**c**) palm + BA, *Ra* = 0.32 μm, *p_H_* = 30 MPa (magnification ×800).

Deformation of the oxides plays an important role in contact phenomena. The oxides are strongly flattened during the friction tests which is the most visible in the case of dry friction conditions (Figure 5a). In the case of lubricated conditions the surface finish was more smooth (Figure 5b,c) than in the case of dry friction. The high volume of open voids was observed, which can be considered as oil reservoirs.

### 3.2. Tribological Performance of Vegetable Oils

Suitable effectiveness of action of the lubricant in reducing frictional resistances in sheet metal forming plays a very important role in SMF. To assess the effectiveness of vegetable lubricants in reducing COF, the value of indicator *δ_s_* was determined according to the relation:

(4) $$\delta_{s}=\frac{\mu_{dry}-\mu_{oil}}{\mu_{dry}}\cdot 100\%$$

where *μ_dry_* and *μ_oil_* are the COFs determined in dry and lubricated conditions, respectively.

**Figure 6:**
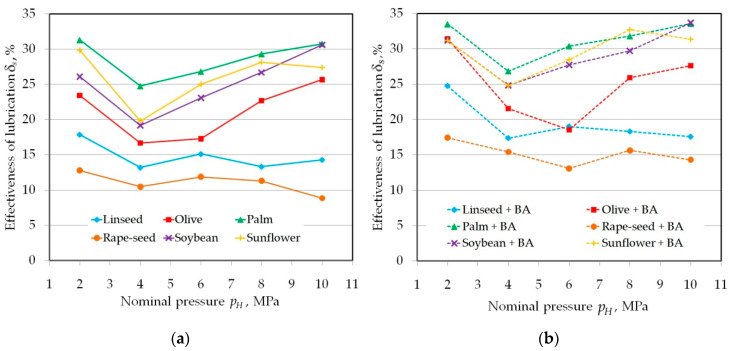
The effectiveness of lubrication on the value of the COF using (**a**) pure vegetable oils and (**b**) vegetable oils with added BA in tests carried out in the range of nominal pressure between 2 and 10 MPa.

The lubrication efficiency of the vegetable oils with and without addition of BA is between 9 and 31% and 13 and 34%, respectively (Figure 6). The character of the effect of oils without BA inclusion can be divided into two groups. In the first group, for linseed and rapeseed oil, the effectiveness was the lowest and decreased with increasing nominal pressure. The remaining vegetable oils showed a distinct local decrease in lubricating properties at a pressure of 4 MPa (Figure 6a) and a further increase in lubricating efficiency. To ensure proper lubrication conditions, the lubricant located in the profile valleys must be subjected to sufficient pressure. It should be noted that the contact area between the strip specimen and the roll for the countersamples with a radius of *R* = 200 N is larger than the contact area of the countersample with a radius of *R* = 10 mm. When the pressure acting on the lubricant is too low, this will not result in an effective boundary lubrication regime.

**Figure 7:**
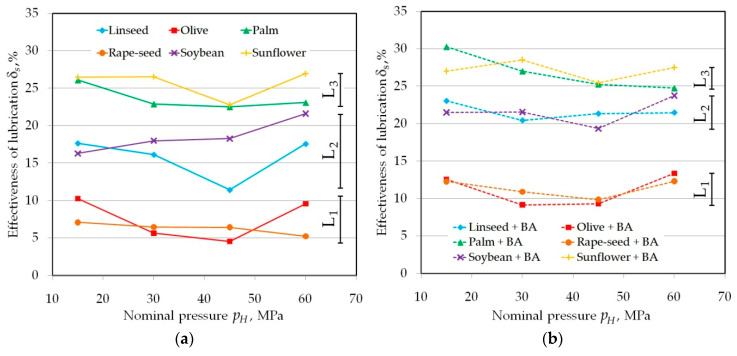
The effectiveness of lubrication using (**a**) pure vegetable oils and (**b**) vegetable oils with added BA in tests carried out under a range of nominal pressure between 15 and 60 MPa for a roll roughness of *Ra* = 0.32 m.

Three clear levels of *δ_s_*-value L1–L3 are visible in Figure 7 regarding tests conducted using countersamples with a radius of *R* = 10 mm and *Ra* = 0.32 μm. Both palm and sunflower oils are the most effective in reducing the COF. This conclusion applies to both pure oils (Figure 7a) and those with the addition of BA (Figure 7b). The interaction of lubricants with the inclusion of BA particles is more uniform across the entire pressure range.

Boric acid, as a hydrate of boric oxide B_2_O_3_, has a strong tendency to form a chemically bonded film on metallic surfaces. The beneficial effect of the addition of boric acid is due to its unique layered crystalline structure. This structure has strong hydrogen bonds within a layer, and weak van der Waals bonds between layers. The structure of H_3_BO_3_ is characterised by low consistency in certain directions of slip, which enables one to conclude that boric acid exhibits anisotropic properties. The shear strength of BA is equal to 23 MPa, and its coefficient of friction is less than 0.02 [55]. The lattice layer structure of H_3_BO_3_ facilitates easy sliding between molecular layers. However, this effect is also dependent on the type of oil in which the BA is dissolved.

**Figure 8:**
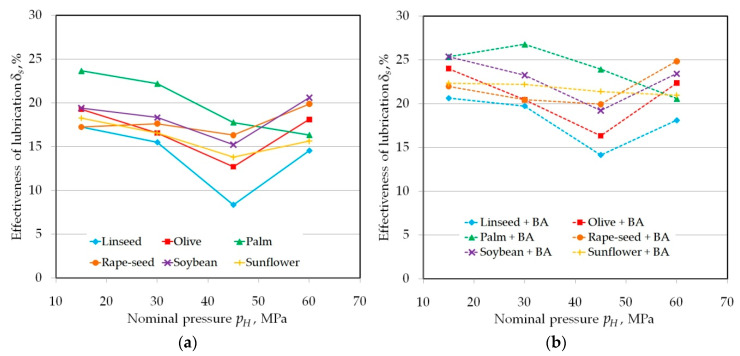
The effectiveness of lubrication using (**a**) pure vegetable oils and (**b**) vegetable oils with added BA in tests carried out under a range of nominal pressure between 15 and 60 MPa for a roll roughness of *Ra* = 0.63 m.

The occurrence of three distinct levels of lubrication efficiency L1–L3 (Figure 7) is the result of the interaction between the lubricant and the asperities of the countersample surface because the increase in its roughness to *Ra* = 0.63 μm (Figure 8) completely disturbed this distinct three-level distribution of curves of lubrication efficiency. A specific decrease in lubrication efficiency is visible at a pressure of 45 MPa. Only pure palm oil exhibits a decrease in effectiveness over the entire range of pressures analysed (Figure 8a). The main mechanisms occurring between metallic surfaces in contact are ploughing of the softer sheet by a harder tool and the effect of lubricant encapsulated in the oil pockets.

**Figure 9:**
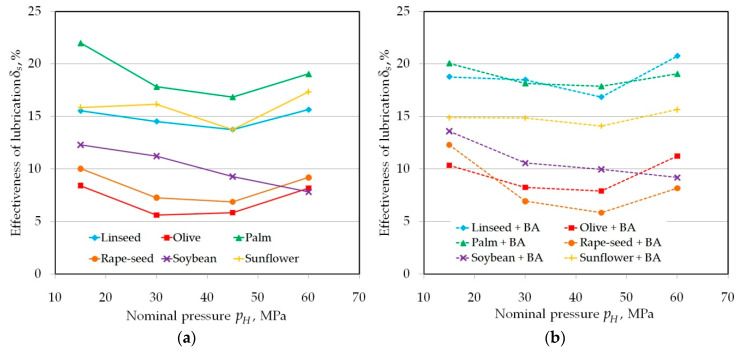
The effectiveness of lubrication using (**a**) pure vegetable oils and (**b**) vegetable oils with added BA in tests carried out under a range of nominal pressure between 15 and 60 MPa for a roll roughness of *Ra* = 1.25 m.

In the case of the countersamples with the highest roughness *Ra* = 1.25 μm (Figure 9), the effectiveness of lubrication does not exceed approximately 22%, while in the case of countersamples with *Ra* = 0.63 μm and *Ra* = 0.32 μm, a decrease of a max. 26% and 31% is observed, respectively.

### 3.3. Effect of Boric Acid Additive

**Figure 10:**
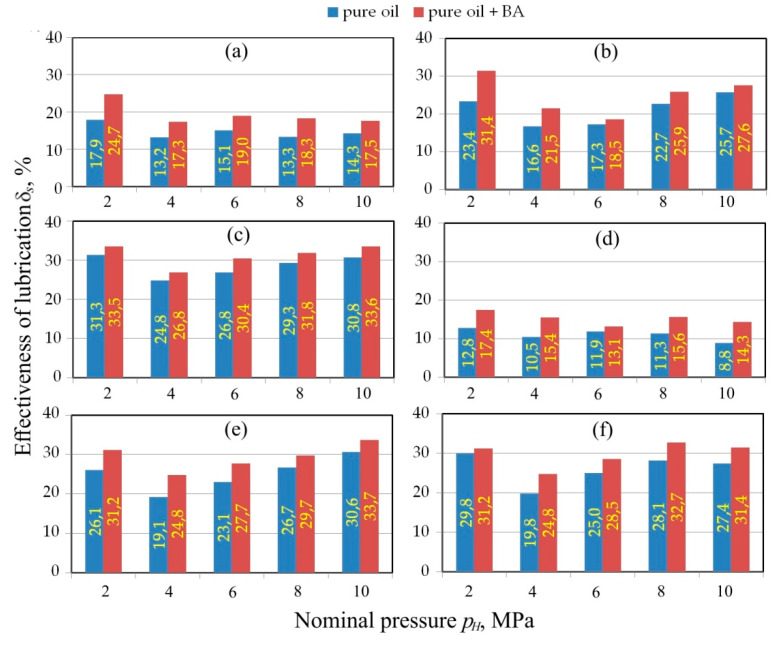
Comparison of the effectiveness of lubrication for tests carried out under the range of nominal pressure between 2 and 10 MPa for different oils: (**a**) linseed, (**b**) olive, (**c**) palm, (**d**) rapeseed, (**e**) soybean, (**f**) sunflower.

During friction tests using rollers with a radius of *R* = 200 mm, the most favourable effect of including BA was observed for rapeseed oil (Figure 10d). The addition of BA in this case increased the lubrication efficiency by approximately 38.9%. In turn, the smallest average increase in lubrication efficiency caused by the addition of boric acid was noted for palm oil (Figure 10c). In this case, in the whole range of nominal pressures, the increase in *δ_s_*-value caused by BA addition is not more than 9.3%.

For all the oils used, there is no clear general relationship between the pressure value and the effect of addition of BA on lubrication efficiency. For olive (Figure 10b), palm (Figure 10c), and soybean (Figure 10e) oils, a clear increase in the lubrication performance is observed when normal pressure exceeds 4 MPa. This relationship applies to both pure oils and oils with added BA and has recently been observed by Erdemir et al. [56] who have shown that the COF of sliding interfaces with a boric acid film decreased with increasing pressure. As was mentioned above, for effective lubrication, vegetable oils and their combinations require the right pressure which is dependent on the interaction between the tool and sheet roughness.

**Figure 11:**
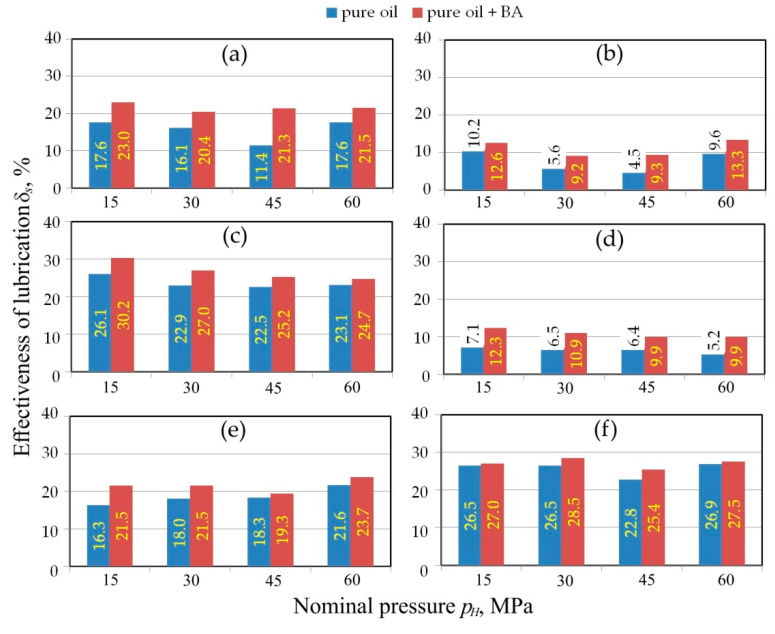
Comparison of the effectiveness of lubrication for tests carried out under the range of nominal pressure between 15 and 60 MPa and roll roughness of *Ra* = 0.32 mm for different oils: (**a**) linseed, (**b**) olive, (**c**) palm, (**d**) rapeseed, (**e**) soybean, (**f**) sunflower.

In the case of tests carried out in the pressure range between 15 and 60 MPa, only soybean oil in both configurations exhibits an increase in lubrication efficiency with an increase in nominal pressure (Figure 11e). For palm oil with addition of BA, this relationship is reversed (Figure 11c). For the whole range of pressures applied, the addition of BA brought the most beneficial effect in the case of rapeseed oil (Figure 11d). The average increase in lubrication efficiency was 80.0%. The results suggest that the addition of BA to rapeseed oil forms a protective layer between the countersample and sheet metal surfaces as the microscale particles are squeezed into the contact interface [57].

### 3.4. Effect of Surface Roughness of the Countersample

**Figure 12:**
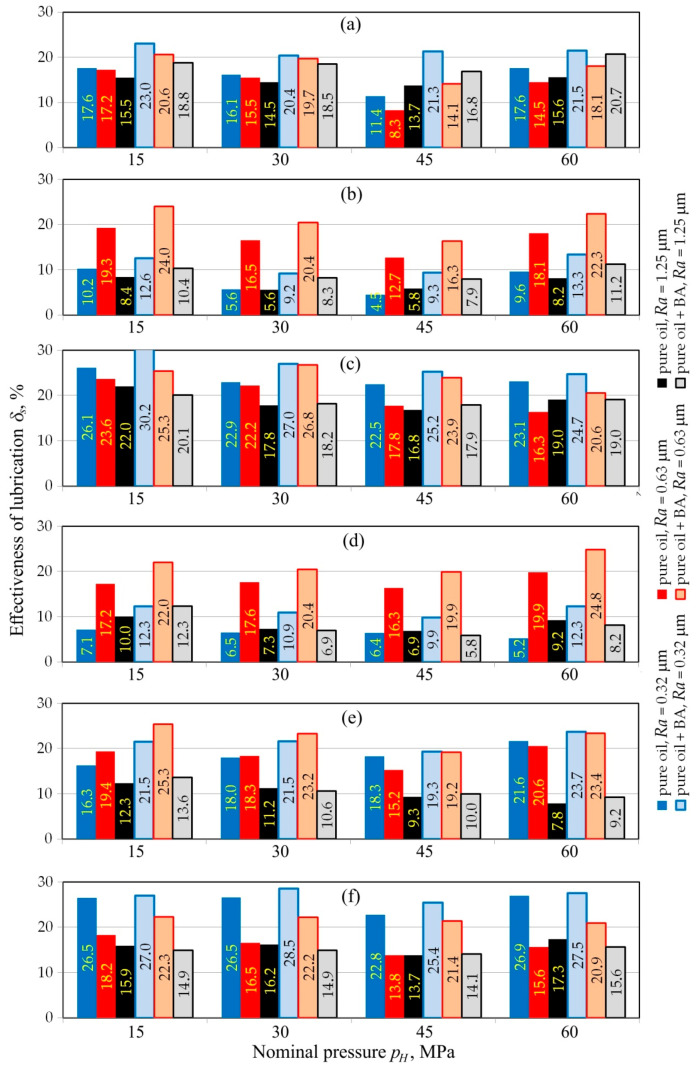
Comparison of the effectiveness of lubrication for tests carried out under the range of nominal pressure between 15 and 60 MPa for different oils: (**a**) linseed, (**b**) olive, (**c**) palm, (**d**) rapeseed, (**e**) soybean, (**f**) sunflower.

Figure 12 shows the effect of the surface roughness of the counter-sample on the lubrication efficiency index *δ_s_*. Four different lubrication mechanisms can be noted, depending on the roughness of the counter-sample and the nominal pressure:

- In the case of palm and sunflower oils, an increase in the surface roughness of the countersample identified by the arithmetic mean value leads to a decrease of the effectiveness of lubrication.
- Rapeseed oil clearly provided the best lubrication efficiency during friction.
- In the range of nominal pressure of *p_H_* =15–30 MPa, increase of countersample roughness caused a decrease in lubrication efficiency for linseed oil. A further increase of nominal pressure value up to *p_H_* = 45–60 MPa showed the worst lubrication efficiency.
- In contrast to linseed oil, the efficiency of soybean oil decreased with increasing pressure.

In most of the friction conditions analysed, an improvement of the lubricating properties of oils was observed following the addition of BA. In most conditions, an increase in the lubrication efficiency of up to 15% is observed when compared to pure oils. However, the addition of the BA to vegetable oils allows it to obtain the beneficial properties of the lubricant. In some cases, the lubrication efficiency was the same for pure and BA-amended oils, even the addition of oil caused a slight reduction in lubrication efficiency. However, due to the small difference between the lubrication efficiency of pure and BA-amended oils, the results may not be statistically significant.

**Figure 13:**
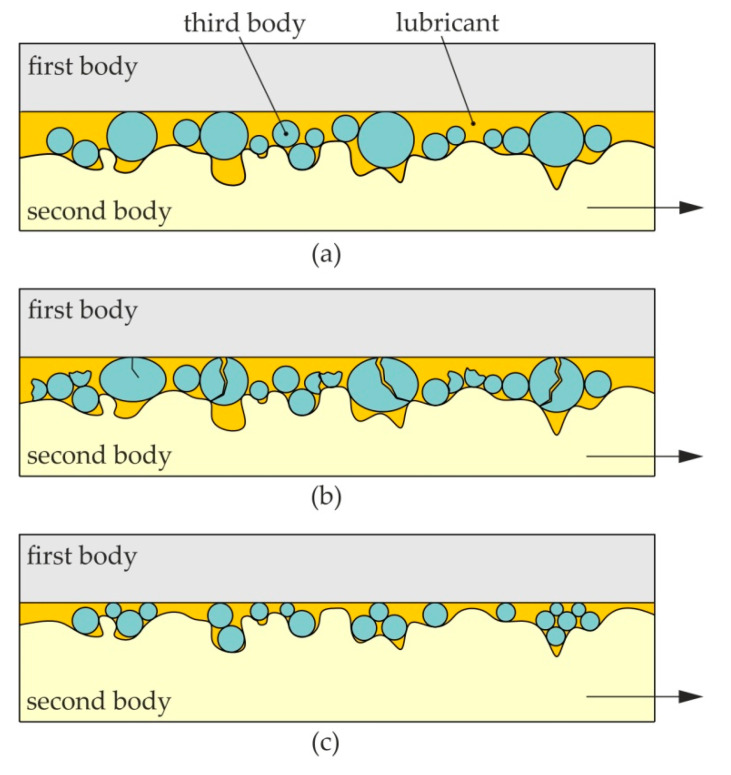
Schematic diagram of the contact interface in the presence of (**a**) large particles, (**b**) high pressure and (**c**) small particles and low-pressure conditions.

BA additives decrease friction by carrying some of the load in valleys between the contacting asperities (Figure 13a). Under high pressure conditions, particles may break down which changes the contact conditions (Figure 13b). If the particles are small enough, they can fill the valleys between the roughness ridges, thereby creating a thin solid film between the contacting surfaces (Figure 13c). In contrast, if the roughness of the tools is too great in relation to the roughness of the sheet, then the mechanism of mechanical action of the vertices of unevenness (i.e., ploughing) is activated. The lubricant will not be able to lower the coefficient of friction under these conditions. BA particles in combination with oils with a high content of fatty acids helped to support the load while being sheared with their low interlayer friction.

## 4. Conclusions

This paper presents the results of experimental investigations on the effectiveness of different edible vegetable oils in pure form and with the addition of BA in reducing the COF in a strip drawing test. In general, all the vegetable oils with additive were shown to be effective in lowering the COF. The lubrication efficiency of the vegetable oils without and with the addition of BA was between 9 and 31% and between 13 and 34%, respectively. The main conclusions drawn are as follows:

- Among deep-drawing quality oils, HDO oil showed the greatest ability to reduce frictional resistance. The high viscosity of this oil suggests that for low pressures it creates conditions for the formation of a lubricant film completely separating the friction surfaces.
- The vegetable oil that provided the greatest reduction in the COF is palm oil in both configurations as a pure oil and with the addition of BA and also when using different radiuses of countersample. Palm oil provides good lubrication under boundary friction due to the presence of long chain fatty acids.
- A mixture of 5 wt.% in palm oil was found to outperform the other lubricant mixtures in the conditions of nominal pressure 15–60 MPa. The effectiveness of lubrication using palm oil was very uniform in the whole range of roughnesses of countersample.
- Hence, the addition of BA to vegetable oils allows one to add beneficial properties to the lubricant. In most conditions an increase in lubrication efficiency of up to 15% is observed when compared to pure oils.
- There are two different effects of oil type on lubrication efficiency. For olive and rapeseed oil, the effectiveness was the lowest and decreased with increasing nominal pressure. The remaining oils showed a distinct local decrease in lubricating properties at a pressure of 4 MPa and a further increase in lubrication efficiency.

## Figures and Tables

**Table 1:** Comparison of properties of mineral oils with vegetable oils.

Property	Mineral Oil	Vegetable Oil
Density, kg·m^−3^ at 20 °C	880	940
Viscosity index	100	100–200
Shear stability	Good	Good
Pour point, °C	−15	−20 to +10
Cloud flow	Poor	Poor
Miscibility with mineral oils	NA	Good
Solubility in water	No	No
Oxidation stability	Good	Mediocre
Hydrolytic stability	Good	Poor
Sludge formation	Good	Poor
Seal swelling tendency	Slight	Slight

Source: With permission from Mobarak et al. [24], Elsevier.

**Table 2:** Selected mechanical properties of DC04 steel sheet.

Yield Stress *R_p_*_0.2_ (MPa)	Ultimate Tensile Stress *R_m_* (MPa)	Elongation *A*_50_ (%)	Strength Coefficient *K* (MPa)	Work Hardening Exponent *n*
185.0	302.5	21.75	481.5	0.185

**Table 3:** Selected surface roughness parameters of sheet tested.

*Sa*, m	*Sq*, m	*Ssk*	*Sku*
1.31	1.53	−0.13	2.10

**Table 4:** Selected surface roughness parameters of countersample with radius *R* = 200 mm.

*Sa*, m	*Ssk*	*Sku*
1.53	−0.014	2.07

**Table 5:** Selected surface roughness parameters of rolls.

*Ra*, m	*Sa*, m	*Sq*, m	*Sp*, m	*Sv*, m	*Sz*, m	*Ssk*	*Sku*	*Sdq*	*Sdr*, %
0.32	0.43	0.56	17.91	52.01	69.92	−0.93	8.43	0.21	2.04
0.63	0.56	0.72	15.60	64.3	22.04	−0.27	4.11	0.18	1.57
1.25	1.34	1.57	1.05	40.51	51.56	−0.29	3.01	0.18	1.57

**Table 6:** Selected properties of synthetic oils used.

Oil	Parameter	Value
MO	kinematic viscosity at 40 °C	43.9 mm^2^·s^−1^
	viscosity index	94
	ignition temperature	232 °C
	flow temperature	−10 °C
DDO	kinematic viscosity at 40 °C	330 mm^2^·s^−1^
	flash point	238 °C
	freezing point	−29 °C
	weld point	500 daN
HDO	density at 20 °C	975 kg·m^−3^
	flash point	277 °C
	viscosity at 40 °C	1157 mm^2^·s^−1^

**Table 7:** Selected properties of the vegetable oils used.

Oil	Density, kg·m^−3^	Kinematic Viscosity at 40 °C, mm^2^·s^−1^
Linseed	880	3.74
Olive	890	4.50
Palm	872	5.69
Rapeseed	883	4.45
Soybean	891	4.05
Sunflower	883	4.45
